# Factors Beyond High-Molecular-Weight Toxin Removal That Might Affect Survival in Comparisons of High-Volume Hemodiafiltration with Hemodialysis

**DOI:** 10.3390/toxins18090365

**Published:** 2026-08-25

**Authors:** John T. Daugirdas

**Affiliations:** Internal Medicine/Nephrology, College of Medicine, University of Illinois at Chicago, 820 S. Wood St., Chicago, IL 60612, USA; jtdaugir@uic.edu

**Keywords:** hemodiafiltration, Kt/V, phosphate, oxalate, urea, intradialytic hypotension, vasoconstrictors, platelets, vesicles, ultrapure dialysate, intravenous saline, plasticizers, microplastics, microparticles

## Abstract

Comparisons of hemodiafiltration with hemodialysis have indicated significantly improved survival when the substitution fluid volume is greater than 23 L per treatment. This survival benefit has been ascribed to better removal of high-molecular-weight uremic toxins. However, with hemodiafiltration, predialysis levels of some high-molecular-weight toxins are only modestly reduced and remain far above levels measured in subjects with normal kidney function. In the present review, the potential role of other factors associated with hemodiafiltration that may beneficially impact survival relative to that seen with hemodialysis is explored. Factors analyzed include removal of relatively small uremic toxins such as urea, phosphate, and oxalate, a reduced incidence of intradialytic hypotension in some studies, possibly related to lower extracorporeal circuit temperature, benefits associated with use of ultrapure dialysis solution, and potential benefits associated with avoidance of thrice-weekly saline infusion for priming, volume replacement, and rinse back. The survival benefit associated with each of these factors may be small, but collectively, they may have a clinical impact.

## 1. Introduction

An apparent disconnect between a marked survival benefit from treatment with hemodiafiltration, and only a modest reduction in predialysis levels of putative high-molecular-weight uremic toxins, was the impetus for the present review. Here we examine additional potential mechanisms which might contribute. 

## 2. High-Molecular-Weight Uremic Toxin Levels

Several relatively large randomized controlled trials (RCTs) examined survival with hemodiafiltration compared to hemodialysis [1,2,3,4,5]. Of the five trials shown in Table 1, two, the ESHOL trial by Maduell et al. [2] and the CONVINCE trial by Blankestijn and colleagues [5], showed a significant survival benefit, while there was no significant survival benefit in the three other trials. The reason proffered for the lack of survival benefit in the three trials which did not show a survival benefit (CONTRAST by Grooteman et al. [1], Turkish by Ok et al. [3] and Frenchie by Morena et al. [4]) was an insufficiently large volume of postdialysis substitution fluid. When the individual data from the first four trials were combined, survival was higher with hemodiafiltration for all enrolled patients, and the benefit was magnified when the substitution fluid volume was 23 L or more per session [6]. In the original RCTs, the volume of substitution fluid was not a prespecified modulator of outcome, and its effect on mortality may have been affected by patient selection bias, similar to the so-called “dose-targeting bias” observed in the HEMO study patients in each randomized dose arm, where survival was found to be increased in patients who had higher achieved dialysis doses [7].

The CONVINCE trial was designed to compare survival between hemodiafiltration and high-flux hemodialysis in patients treated with a high volume of postdilution substitution fluid in the hemodiafiltration arm. It focused on enrolling patients who would be able to achieve large substitution fluid volumes. The CONVINCE trial [5] results were positive, supporting a survival benefit of hemodiafiltration over hemodialysis.

The putative mechanism of increased survival with hemodiafiltration is an increased removal of high-molecular-weight uremic toxins in the molecular weight range of 11 to 45 kilodaltons. Indeed, the reduction ratio of such toxins with hemodiafiltration substantially exceeds that obtained with high-flux hemodialysis [8]. However, for many of these toxins, the postdialysis rebound is quite marked, and because of rebound, relatively small volumes of distribution, and for some toxins, substantial extrarenal clearances, the predialysis levels with hemodiafiltration are only marginally lower than those observed with high-flux hemodialysis.

**Figure 1:**
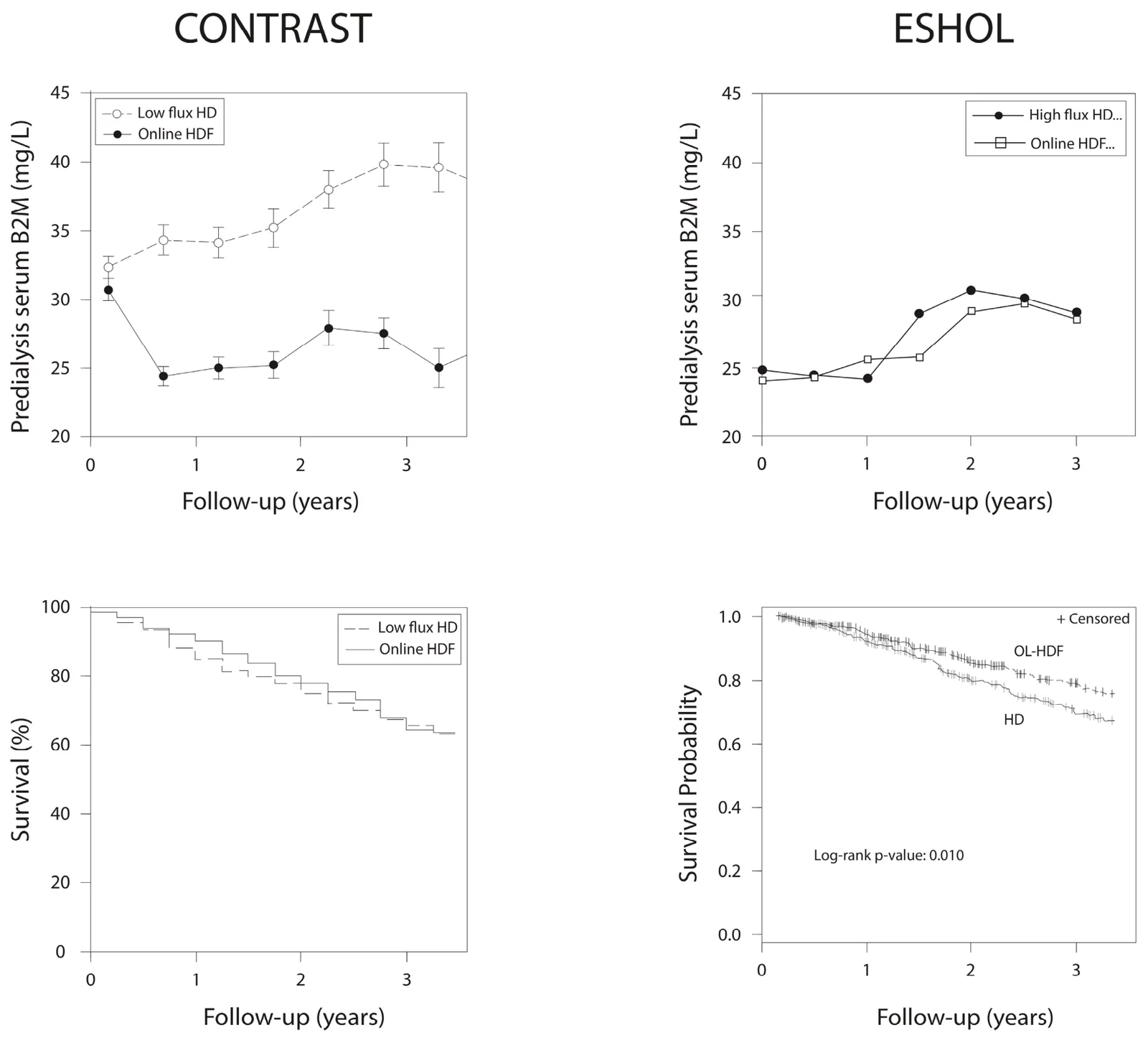
Divergence in outcomes between differences (or lack thereof) in predialysis serum beta-2-microglobulin (top panels) and differences in mortality (bottom panels) as reported in two randomized trials: the CONTRAST Study (Grooteman et al. [1]) and the ESHOL study (Maduell et al. [2]). Data truncated to the first 3 years of follow-up. ESHOL study baseline and follow-up predialysis beta-2-microglobulin data are taken from the ESHOL published supplementary data file. Left panels and bottom right panel reproduced with permission from Grooteman MP et al., *J. Am. Soc. Nephrol.* 2012, 23, 1087–1096, and Maduell F et al., *J. Am. Soc. Nephrol.* 2013, 24, 487–497, respectively.

The predialysis serum beta-2-microglobulin levels were reported in 4 of the 5 major randomized comparisons of hemodiafiltration. The values are given in Table 1. During follow-up, marked differences in predialysis serum beta-2-microglobulin levels between the hemodiafiltration and hemodialysis groups were seen only in the CONTRAST trial, where the comparator hemodialysis group was treated with low-flux dialyzer membranes. In the three other trials in which predialysis serum beta-2-microglobulin levels were measured, there was minimal to no difference between the levels during follow-up. Figure 1 shows another conundrum. In the one trial (of the initial four) in which a 25% survival benefit was found, the ESHOL study [2], there was minimal difference in predialysis serum beta-2-microglobulin levels during follow-up, whereas in the CONTRAST study [1], which used a low-flux hemodialysis comparator, predialysis serum beta-2-microglobulin levels were reduced substantially with hemodiafiltration; despite this, the hemodiafiltration and hemodialysis survival curves were nearly identical.

Another complexity relating to the high-molecular-weight toxin removal hypothesis explaining the survival benefits with hemodiafiltration has to do with the effects of substitution fluid volume on predialysis serum beta-2-microglobulin levels. In the post hoc analyses that were done of these initial four trials, where a survival benefit was found with higher substitution fluid volumes, the impact of substitution fluid volume on predialysis serum levels of beta-2-microglobulin or other putative high-molecular-weight uremic toxins was not reported. When beta-2-microglobulin removal during hemodiafiltration is kinetically modeled, when comparing substitution fluid volumes of 15 L with 25 L per 4 h treatment, the serum beta-2-microglobulin reduction ratio is modeled to increase from 72% to 79%, but the impact of the higher substitution fluid volume on predialysis serum beta-2-microglobulin is modeled to be only about 4% (see Figure 4 in reference [9] by Ward and Daugirdas).

The relative benefits of solute removal with hemodiafiltration compared with hemodialysis increase as the dialyzer permeability for the solute in question decreases [9]. Beta-2-microglobulin, with a molecular weight of 12 kilodaltons, is in the lower range of putative high-molecular-weight uremic toxins [10]. For higher-molecular-weight toxins, for example kappa and lambda free light chains, with molecular weights of 22.5 and 45 kilodaltons, respectively, the enhanced solute removal with hemodiafiltration compared with hemodialysis is magnified [11]. However, when predialysis values of such very large putative uremic toxins have been measured, the difference in predialysis serum levels with hemodiafiltration vs. hemodialysis is not large [8,9,10,11,12] and predialysis levels remain many times higher than serum values in normal subjects.

When assessing dialysis adequacy in terms of urea, we focus on the urea reduction ratio rather than on any changes in predialysis serum urea level. This is partly due to confounding by marked differences among patients in their rates of urea generation, which is a reflection of protein intake and transitively, of food intake, reflecting overall health. Solute reduction ratios might be as important as or more important than the predialysis serum levels; still, the impressive differences in mortality seen in some studies comparing hemodiafiltration with hemodialysis with only small differences in predialysis serum levels of large toxins, leaves open the possibility that additional mechanisms may be at play. As an example, for beta-2-microglobulin, a typical serum range in subjects with normal kidney function is 0.7 to 1.7 mg/L (average 1.2 mg/L). Wathanavasin et al., in a meta-analysis [12], found that average predialysis serum beta-2-microglobulin in patients treated with hemodiafiltration was 23.8 mg/L, and that the average difference in predialysis level with hemodiafiltration compared with high-flux hemodialysis was only 2.65 mg/L (11%) lower. Thus, baseline values of serum beta-2-microglobulin in patients treated with hemodiafiltration are still 20-fold higher than levels in patients with normal kidney function. One needs to explain how an 11% reduction in serum beta-2-microglobulin levels from such a markedly elevated baseline value, could lead to a substantial decrease in mortality. A partial counterargument might be based on comparing the predialysis serum beta-2-microglobulin lowering effect of high-volume hemodiafiltration with that of residual kidney function. Kinetic modeling suggests that high-volume hemodiafiltration with a postdilution substitution fluid infusion rate of 100 mL/min over 4 h should lower predialysis serum beta-2-microglobulin by the same amount as 1.5 mL/min residual kidney glomerular filtration (GFR) [9]. Even relatively small amounts of residual kidney GFR are associated with markedly improved survival [13]. Whether or not 1.5 mL/min of such “equivalent” residual kidney GFR can effect a reduction in mortality of 20–25%, (especially in the absence of the benefit of reduced ultrafiltration that is normally seen in patients with residual kidney function) is a question that needs further study.

## 3. Enhanced Removal of Low-Molecular-Weight Toxins 

**Figure 2:**
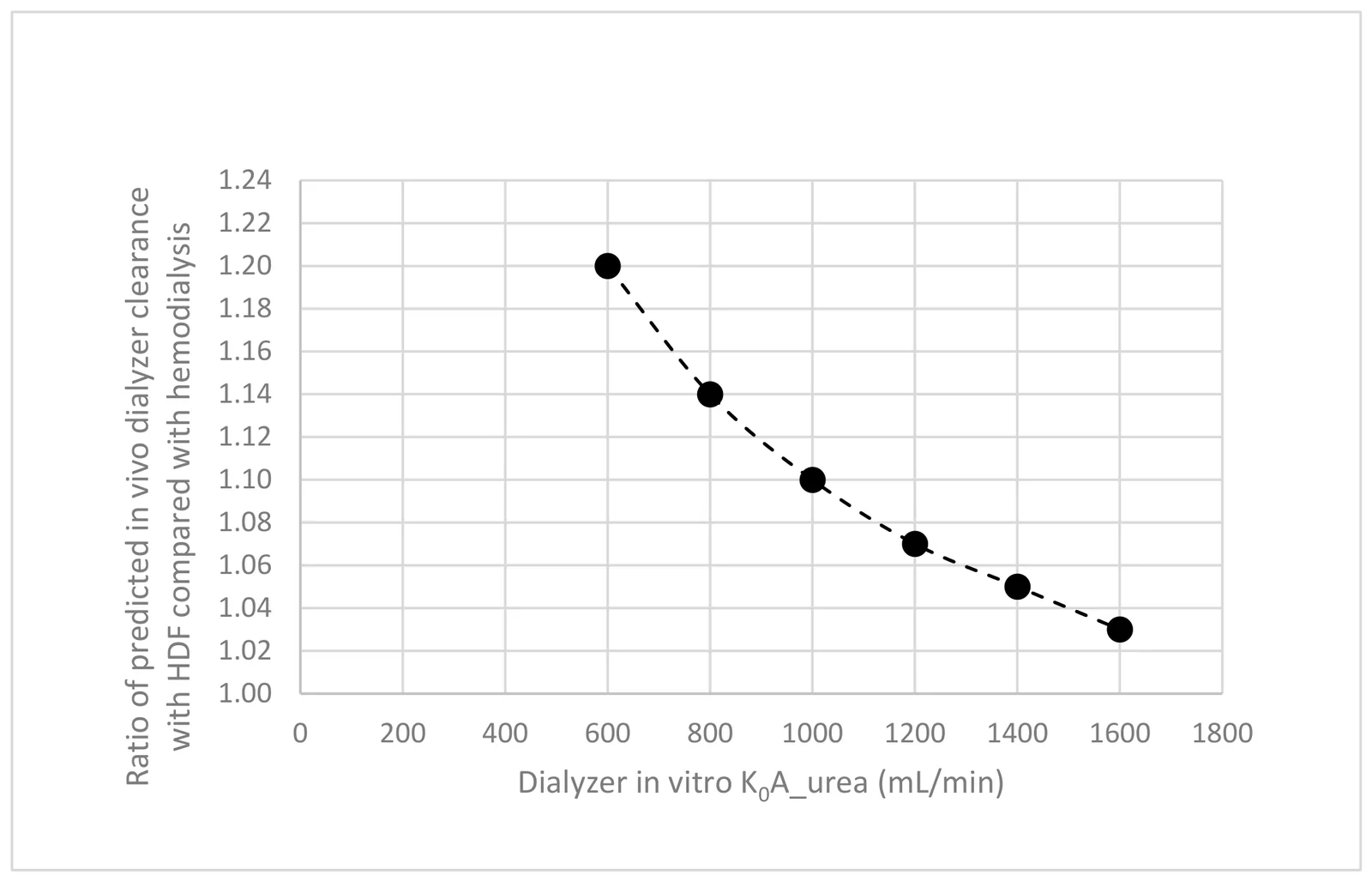
Ratio of modeled in vivo dialyzer urea clearance with hemodiafiltration to that with hemodialysis, as a function of dialyzer solute permeability to urea, expressed as in vitro K_0_A urea in mL/min. The diffusive clearances were calculated using the Michaels equation, assuming that in vivo K_0_A urea was 0.537 times the industry-reported in vitro value, blood flow rate was 350 mL/min and dialysate flow plus substitution fluid flow rate was 500 mL/min. The EuDial equations were used to compute the convective clearance. The assumption was a substitution fluid rate in postdilution mode of 100 mL/min.

**Urea:** As a general principle, the additive amount of solute clearance with hemodiafiltration compared with hemodialysis will vary inversely with dialyzer membrane permeability for that solute (which can be expressed as the dialyzer mass transfer area coefficient). For urea, a molecule having a molecular weight of 60 daltons, dialyzer permeability is quite high, and so the increased clearance associated with adding 100 mL/min of postdilution hemodiafiltration is not large. The added clearance due to hemodiafiltration will diminish as the in vitro K_0_A urea of the dialyzer being used increases (Figure 2). For example, if one models dialyzer urea clearance with or without hemodiafiltration, assuming a blood flow rate of 350 mL/min, urea clearance would increase by 7% with hemodiafiltration if the dialyzer used has an in vitro K_0_A urea of 1200 mL/min, but by only 3.5% if a very high-efficiency dialyzer is used, e.g., with an in vitro K_0_A urea of 1600 mL/min.

Does hemodiafiltration cause a meaningful increase in Kt/V urea? Either single-pool (sp) or equilibrated (e) Kt/V urea values were reported in each of the five randomized comparisons of hemodiafiltration with hemodialysis listed in Table 1, and the trial-reported baseline and follow-up Kt/V values are shown in Table 2.

As can be seen, there is an improvement in Kt/V with hemodiafiltration in 3/5 trials (with minimal improvement in the Morena et al. trial), ranging from 8 to 16%. However, in the comparator hemodialysis groups, the mean spKt/V averaged 1.38 to 1.61. Can an 8–16% increase in Kt/V from such a relatively high baseline level of spKt/V explain some of the survival benefit of hemodiafiltration? The only randomized study exploring adequacy of hemodialysis in terms of Kt/V urea as a primary metric was the NIH-funded HEMO study [14]. That study found no difference in survival in patients being dialyzed to a spKt/V of approximately 1.3 compared to patients being dialyzed to a spKt/V of approximately 1.7. However, when results were analyzed separately for men and women, in women, the higher spKt/V value was associated with improved survival [14,15]. Thus, it remains possible that the small increase in spKt/V seen with hemodiafiltration might account for some of the survival benefit seen. A counterargument would be that in the main randomized comparisons of hemodiafiltration with hemodialysis, there was no strong hint that the survival benefit of hemodiafiltration was larger in women compared with men.

**Figure 3:**
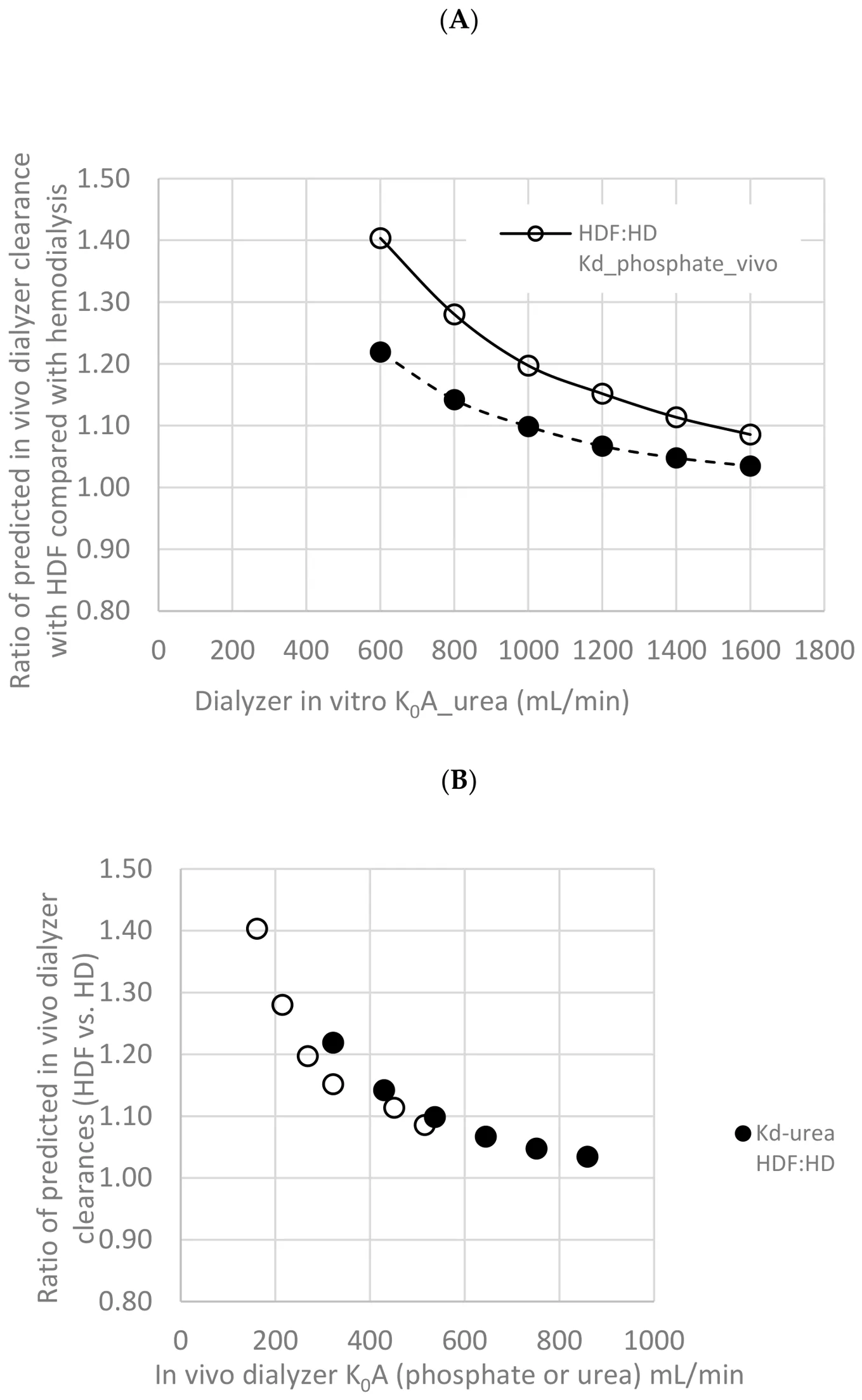
(**A**) shows the ratio of modeled in vivo dialyzer clearance of phosphate (open circles) or urea (solid circles) as a function of dialyzer solute permeability, expressed as in vitro K_0_A urea. (**B**) shows the same modeled data as in (**A**), the ratio of predicted in vivo dialyzer clearance with hemodiafiltration vs. hemodialysis for urea (solid circles) and phosphate (open circle), but here data are plotted vs. predicted dialyzer in vivo K_0_A for either phosphate or urea. It is assumed that the ratio of K_0_A phosphate to K_0_A urea is 0.55, based on data by Bhimani et al. [18].

**Figure 4:**
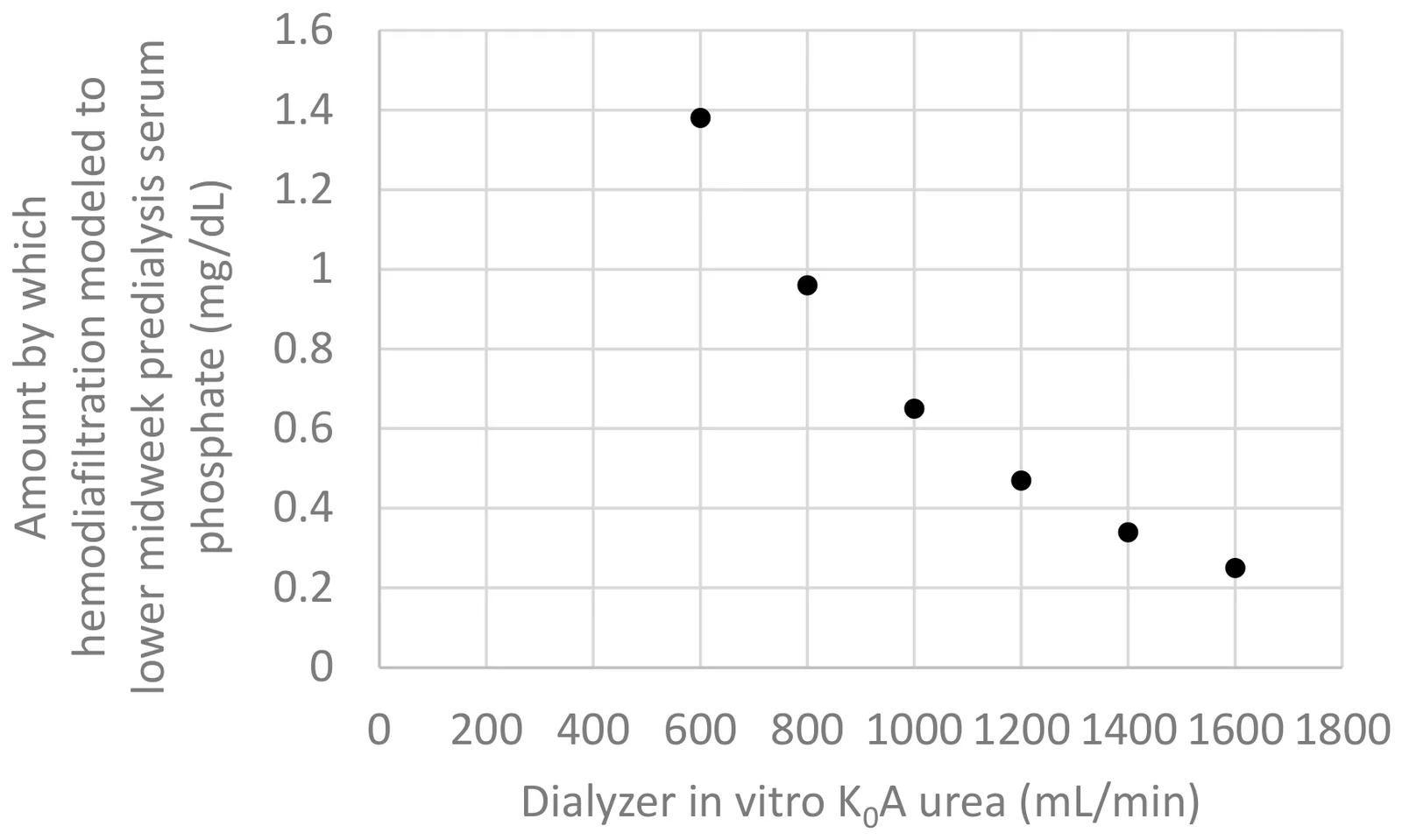
Predicted difference (amount lower) in midweek predialysis serum phosphate with hemodiafiltration vs. hemodialysis as a function of dialyzer solute permeability (as in vitro K_0_A urea). Kinetic modeling assumptions regarding dialysis treatment and phosphate intake are as previously described [16].

**Phosphate:** Because the dialyzer solute permeability to phosphate is substantially lower than that to urea [16,17,18], due to both a higher molecular weight (95 vs. 60 daltons) and the presence of a negative charge, one would expect that the clearance benefit of hemodiafiltration for phosphate would be higher than that for urea. Kinetic modeling equations suggest that for a moderate efficiency dialyzer (in vitro K_0_A urea 1200 mL/min) or for a very high efficiency dialyzer (in vitro K_0_A urea 1600 mL/min), at a blood flow rate of 350 mL/min and a postdilution substitution fluid flow rate of 100 mL/min, the estimated in vivo dialyzer clearance for phosphate would be 17% and 10% higher, respectively, with hemodiafiltration than with hemodialysis. These clearance increases are higher than the 7% and 3.5% increases predicted with hemodiafiltration for urea [16,17], as discussed above. With regard to impact on predialysis serum phosphate, kinetic modeling [17] predicts that when using dialyzers of these two efficiency levels, midweek predialysis serum phosphate would be lower with hemodiafiltration by 0.5 or 0.3 mg/dL, respectively (Figure 3 and Figure 4). Predialysis serum phosphate level is controlled not only by the amount of dialytic phosphate removal, but also by the amount of dietary phosphate ingestion, the amount of phosphate binders being taken, and the level of residual kidney function [16]. Relatively small changes in levels of any of these ancillary parameters can easily reduce or eliminate the serum phosphate lowering benefit of hemodiafiltration [16]. In a meta-analysis of randomized trials comparing hemodiafiltration with low-flux or high-flux hemodialysis, with hemodiafiltration the mean predialysis serum phosphate was lower by 0.28 mg/dL [12], similar to the value predicted by kinetic modeling.

Could the relatively modest reduction in serum phosphate with hemodiafiltration contribute to a survival benefit? In a large cross-sectional analysis, a higher predialysis serum phosphate (greater than 6.0 mg/dL) was indeed related to mortality [19]. Thus, hemodiafiltration might be associated with increased survival if it is associated with a reduction in serum phosphate levels, but this might only be found when baseline predialylsis levels are above 6.0 mg/dL.

**Oxalate:** The serum levels of oxalate are normally around 2 micromol/L, while in hemodialysis patients, predialysis serum levels are fifteen- to thirty-fold higher [20,21,22]. Higher quartiles of plasma oxalate levels in dialysis patients have been associated with increased risk of cardiovascular events and sudden cardiac death [21]. Dialyzer membrane permeability to oxalate, a negatively charged ion with a molecular weight of 88 daltons which has a similar ionic radius to phosphate, should be similar to that of phosphate, and so hemodiafiltration should be able to enhance oxalate removal, especially when relatively low-efficiency dialyzer membranes are used. However, with very high efficiency dialyzers, a clearance benefit of hemodiafiltration may not be seen. For example, Ermer et al., in a carefully designed crossover trial, compared oxalate removal with hemodiafiltration vs. hemodialysis. Both treatments were given with 2.2 m^2^ very-high-efficiency dialyzers. The investigators found no difference in either the intradialysis plasma oxalate reduction ratio nor in predialysis serum oxalate levels when measured at the end of 2 weeks on each treatment modality [20]. The results of Ermer were in contrast to data from earlier studies using dialyzers of substantially lower solute permeability, in which oxalate removal by hemodiafiltration was superior to that obtained with hemodialysis [22,23].

To summarize the findings relating to low-molecular-weight uremic toxins, hemodiafiltration does increase clearance of urea and phosphate, and probably also of oxalate, but the increased clearance with hemodiafiltration is modest in amount and dwindles as dialyzers of high solute permeability are used. Dialyzers of very high permeability are not universally used. For dialyzers with an in vitro K_0_A urea of 1200 mL/min or less, at typical adult blood flow rates of 350 mL/min, a meaningful increase in clearance of lower-molecular-weight solutes can be anticipated. However, the clinical benefit of increased clearance of low-molecular-weight uremic toxins with hemodiafiltration is likely to be small.

## 4. Intradialytic Hypotension

Intradialytic hypotension, when defined strictly as systolic blood pressure less than 90 mm Hg (or less than 100 mm Hg in patients with higher baseline systolic pressures), occurs in approximately 10% of hemodialysis sessions [24,25]. Most of these episodes are asymptomatic [26]. In patients with frequent intradialytic hypotension episodes, risk of mortality is increased [27,28] and the risk association is higher with episodes occurring during the initial half of a dialysis session [28]. It has long been known that thermal energy transfer during dialysis to the patient is associated with risk of dialysis hypotension. The putative physiological mechanisms are as follows [29,30,31]: During dialysis with ultrafiltration, as fluid is removed, there is compensatory constriction of the arterioles, including those in the skin, to help maintain blood pressure and cardiac output. This reduces skin blood flow, reducing the rate of radiation of body heat to the environment, and ultimately increasing core body temperature. The latter activates vasodilatory responses, which then contribute to a susceptibility to hypotension. This reduced body heat radiation due to skin vasoconstriction can be compensated for by slightly reducing the temperature of blood flowing through the extracorporeal circuit. In support of this hypothesis, use of cooler dialysate has consistently been associated with a reduced risk of dialysis hypotension in most studies [29,30,31,32,33] with one notable exception being the large cluster randomized MyTemp trial [34].

Because cool dialysate use has been associated with a lower incidence of dialysis hypotension, and dialysis hypotension frequency has been associated with increased mortality risk, studies evaluating any link between cool dialysate use and survival are of interest. Unfortunately, only a handful of such studies have been done. An observational study from Taiwan, based on results from 910 patients, 165 of whom were being dialyzed with cool dialysate (<35.5 °C), found that those patients receiving cool dialysate had a lower mortality risk, including a lower cardiovascular mortality risk [35]. This was not, however, observed by Zoccali et al., based on data from more than 8000 patients [36]. That study did find a very modest 2.5% reduction in dialysis hypotension episodes with cool dialysate, but no impact of cool dialysate use on mortality. Finally, the MyTemp cluster randomized trial [34], which failed to find even a trend for reduced incidence of dialysis hypotension with cool dialysate use, also failed to find any survival benefit associated with use of a slightly cooler dialysis solution.

What does discussion of thermal energy transfer have to do with hemodiafiltration? The improved hemodynamic stability observed with isolated ultrafiltration, hemofiltration, and hemodiafiltration has been shown to be associated with thermal balance. The effect of hemodiafiltration on cooling of the extracorporeal circuit was stronger prior to the adoption of online hemodiafiltration, because initially, substitution fluid was infused from a container usually kept at room temperature [37]. With on-line hemodiafiltration the impact on cooling of the extracorporeal blood circuit is not clear. In one study, extracorporeal blood cooling was seen when substitution fluid was prepared online from prewarmed dialysate [38], though in another study, online postdilution hemodiafiltration did not result in a negative thermal energy balance compared to conventional hemodialysis [39]. Pinney et al. [40] compared blood pressures during hemodiafiltration vs. during cool hemodialysis, and found that the incidence of hypotension was similar. Buchanan and colleagues [41] found no differences in hemodynamic responses when comparing hemodiafiltration with slightly cooled hemodialysis.

Rootjes et al., in the HOLLANT study, compared intradialytic hypotensive episodes with conventional vs. cooled hemodialysis and also with low-volume vs. high-volume hemodiafiltration. They used a balanced crossover design in which blood pressure was measured every 15 min during each of these four treatment variations [42]. The incidence of dialysis hypotension was reduced with cool dialysate use during hemodialysis, and was also lower with high-volume hemodiafiltration compared to low-volume hemodiafiltration. The beneficial effect on hypotension incidence of high-volume hemodiafiltration could not be explained by a temperature effect, as high-volume hemodiafiltration had no differential effect on core body temperature. In a follow-up analysis by Liu et al., [43] blood levels of a variety of endothelial- and platelet-derived vesicles were measured before and after each treatment modality. Circulating levels of platelet-derived vesicles were higher at the end of both low- and high-volume hemodiafiltration treatments compared to the hemodialysis sessions. Platelet-derived vesicles contain thromboxane and serotonin, both vasoconstrictors, and one hypothesis might be that the increased hydrodynamic stress on blood components during hemodiafiltration leads to greater release of such vasoconstrictor-containing vesicles, which might result in a lower incidence of intradialytic hypotension. One piece of data that does not fit this hypothesis was the lower incidence of hypotension during high-volume hemodiafiltration compared with low-volume hemodiafiltration treatments, but no difference in elevated levels of platelet-derived vesicles between high- and low-volume sessions [43]. Liu et al. [43] also measured blood levels of endothelial- and cardiomyocyte-derived vesicles and found these to be increased to a lesser extent with hemodiafiltration than with standard (non-cooled) hemodialysis, and speculated that this reflected less cardiovascular injury associated with hemodiafiltration compared to hemodialysis, and that this might explain fewer episodes of intradialytic hypotension with hemodiafiltration.

In the five major randomized comparisons of hemodiafiltration with hemodialysis discussed above, thermal balance was not documented. The incidence of intradialytic hypotension was not specifically documented either, with the exception of the ESHOL and Frenchie trials. In ESHOL, the incidence of dialysis hypotension was markedly reduced in the hemodiafiltration arm vs. the hemodialysis arm: 679 episodes per 100 patient years vs. 938, respectively, *p* < 0.001. In Frenchie, the incidence of asymptomatic hypotension was lower in the hemodiafiltration arm, but the magnitude of the difference was modest.

To summarize this section, the dose–response relationship between convective volume and survival in hemodiafiltration trials is consistent with a magnified putative thermal benefit at higher substitution volumes. However, the absence of a mortality benefit in large cool-dialysate trials (e.g., the Mytemp study) and the lack of any long-term study that isolates thermal balance from convective clearance with hemodiafiltration, suggest that this is a hypothesis-generating concept only. At the present state of knowledge, it is likely that thermal effects explain only part of the survival advantage of hemodiafiltration. The HOLLANT study, while confirming greater hemodynamic stability with high-volume hemodiafiltration, called the thermal balance mechanism into question, and at the same time raised other potential non-thermal hypotheses for increased blood pressure stability during hemodiafiltration.

## 5. Use of Ultrapure Hemodialysis Fluid

In most randomized controlled trials comparing online hemodiafiltration to hemodialysis, ultrapure dialysate (very low endotoxin and bacteria levels, typically <0.03 EU/mL and 0.1 colony-forming units (CFU) per mL, respectively), often produced online or with advanced purification systems, is used in both arms. This separates the effect of increased convection with hemodiafiltration from potential benefits of ultrapure dialysate use. In real-world observational data, however, many hemodialysis patients still receive standard (non-ultrapure) dialysate, which is associated with a higher level of systemic inflammation compared to when ultrapure dialysate is used. Even low-level endotoxin exposure in standard dialysate acts as a chronic inflammatory stimulus, elevating C-reactive protein (CRP) and interleukin-6 (IL-6). Ultrapure dialysate minimizes backfiltration of endotoxins and other bacterial fragments across the dialyzer membrane. Switching patients from standard to ultrapure dialysate while they remain on hemodialysis produces clinically meaningful reductions in inflammatory markers (CRP and IL-6) and predialysis serum levels of beta-2-microglobulin [44,45,46,47,48]. On switching to ultrapure dialysate use, the decreases in predialysis serum beta-2-microglobulin can be quite marked, despite no change in convective clearance. The presumed mechanism is a reduction in generation of beta-2-microglobulin due to decreased inflammation.

Despite some of the preliminary evidence cited above that focused on serum levels of inflammatory mediators, whether use of ultrapure dialysate results in a survival benefit while remaining on the hemodialysis modality is not a question which has been definitively answered. One large (131,000 patients) observational study from Japan by Hasegawa et al. [49] found that mortality was significantly associated with facility dialysate endotoxin levels, but the strongest association was with dialysate endotoxin levels above the acceptable minimum limit of 0.05 EU/mL. Mortality rate was 67 per 1000 person-years when endotoxin levels were <0.001 EU/mL, consistent with “ultrapure” dialysate, while the average death rates were 72–75 per 1000 person-years in facilities where endotoxin levels were between 0.001 and 0.05 EU/mL. A relatively large randomized trial compared fatal/non-fatal cardiovascular evenets l in low- vs. high-flux dialysis and use of ultrapure vs. standard dialysis solution using a two-by-two factorial design [50]. Use of ultrapure dialysate was not associated with a significant reduction in cardiovascular events, with a mean mortality hazard ratio of 0.90, *p* = 0.60.

To summarize this section, use of ultrapure dialysate has been shown to substantially lower levels of inflammatory markers and serum beta-2-microglobulin, and this might account for part of the survival benefit ascribed to hemodiafiltration in observational data, where non-ultrapure dialysate is often used for hemodialysis; however, this cannot explain benefits found in randomized comparisons of hemodiafiltration with hemodialysis in which ultrapure dialysate was used in both treatment arms. As use of ultrapure dialysate for hemodialysis becomes a global standard, the additive benefit of ultrapure dialysate use with hemodiafiltration becomes less relevant. However, when comparing hemodiafiltration with hemodialysis in observational data where ultrapure dialysate was not used for the hemodialysis treatments, some of the benefits of hemodiafiltration with regard to inflammatory mediator reduction may have been due to the use of ultrapure dialysate in the hemodiafiltration group.

## 6. Potential Benefits of Not Using Saline Infusion for Dialyzer Priming, Correction of Volume Depletion, and Rinse Back

In conventional hemodialysis, the dialyzer and extracorporeal circuit are typically rinsed/primed with approximately 300–1000 mL of 0.9% normal saline. This flushes out air, residual sterilants (e.g., ethylene oxide, formaldehyde, or glycerin), and manufacturing residues while priming the blood lines and dialyzer fibers. The actual priming volume needed just to fill the circuit (dialyzer blood compartment + tubing) is smaller—typically 200–400 mL total, depending on the dialyzer size and line set (e.g., many dialyzers have an internal blood volume of ~100–150 mL). However, sometimes a larger volume (usually from a 1 L saline bag) is used to ensure thorough rinsing, with the last ~500 mL sometimes heparinized (e.g., 1000 U heparin) to “coat the membrane” in an attempt prevent clotting. At the end of a dialysis session, a separate rinse back (return of blood to the patient) is done, using an additional 200–300 mL saline. There is no single universal volume mandated by major international bodies such as KDOQI or KDIGO for new dialyzers. Manufacturers commonly instruct using a 1 L saline bag and priming until the circuit is filled/clear (often ~300–500 mL minimum, with flow ≤ 150 mL/min to avoid air-locking). Thorough rinsing is emphasized to minimize dialyzer reactions and ensure biocompatibility.

For online hemodiafiltration, the rinse/priming fluid is typically the machine-generated ultrapure substitution fluid (produced online from dialysate via a sterilizing ultrafilter) instead of bagged saline. This eliminates the need for external saline bags, reduces costs, allows more precise volume control by the machine, and uses fluid with slightly lower sodium content than 0.9% saline (potentially reducing sodium loading).

There is no evidence that the volume of rinse fluid typically used with online hemodiafiltration is higher compared with standard hemodialysis. The procedure and volumes are analogous, although it is easier to economically increase the volume of rinsing fluid with hemodiafiltration, potentially enhancing removal of microbubbles of air from the extracorporeal circuit, as well as leachable substances present in the dialyzer left over from the manufacturing process. If a single 1 L bag of saline is used for a dialysis treatment, 500 mL is used for priming and several hundred mL are infused to treat intradialytic hypovolemia, the amount of saline left for rinse back at the end of dialysis may be limited. A study by Matos et al. tried to determine the optimum volume of saline to use at the end of dialysis to ensure maximum reinfusion of red blood cells resident in the extracorporeal circuit at the end of dialysis, while limiting the risk of fluid overload [51]. Their conclusion was that the optimum volume of rinse-back fluid was 380 mL. Use of ultrafiltered dialysate for the rinse-back fluid removes the volume constraint associated with use of bagged saline, and allows use of an optimum rinse-back volume unrelated to how much fluid was infused during earlier parts of the treatment. Thus, there are both cost savings and environmental benefit when switching to online fluid for priming and rinse back and avoiding the use of bagged intravenous saline [52,53,54].

**Microplastics and nanoplastics:** There is emerging evidence confirming the presence of microplastics, nanoplastics, and other contaminants including chemical leachates like DEHP (Di(2-ethylhexyl) phthalate) in bags of saline and related infusion fluids, with direct delivery into the bloodstream during intravenous administration [55,56]. However, direct clinical evidence linking routine intravenous saline-bag microplastics to adverse patient outcomes remains limited. Research highlights concerns rather than proven causation.

In summary, for this section, the ability with hemodiafiltration to use online, highly purified solution for initial priming and rinsing of dialyzer, infusion to correct intravascular volume contraction, and rinse back, offers cost-savings and reduction in environmental waste. Health benefits of avoiding routine intravenous saline exposure remain speculative.

## 7. Comparison of Hemodiafiltration with So-Called “Expanded Hemodialysis” (HDx)

Expanded hemodialysis (HDx) using medium cut-off (MCO) membranes also significantly enhances middle molecule clearance beyond that achieved with conventional high-flux hemodialysis. With HDx this occurs through internal convection within the dialyzer plus optimized membrane pore size. Zhao et al. [57], in a meta-analysis of 18 studies, demonstrated that HDx achieves superior removal of larger middle molecules such as kappa and lambda free light chains compared to hemodiafiltration, with comparable albumin loss and overall safety. In contrast, Kuo et al. [58] and Lukkanatiktikul and colleagues [59] suggested that hemodiafiltration may offer advantages in terms of slightly higher reduction ratios of certain high-molecular-weight middle molecules.

The MOTheR HDx trial was a multicenter open-label, prospective 1:1 randomized parallel-group controlled trial, comparing HDx with hemodiafiltration. The trial was designed to evaluate non-inferiority of HDx treatment vs. hemodiafiltration. Five hundred thirty-three patients were enrolled, with mean follow-up of 20–24 months.

The trial results have yet to be published in a peer-reviewed journal, but have been reported in abstract form at the European Renal Association Congress in 2026 [60]. The preliminary results suggested that HDx was non-inferior to online hemodiafiltration for a primary composite endpoint of all-cause mortality and major cardiovascular events (IRR 0.87, 95% CI 0.63–1.19; *p* = 0.011 for non-inferiority). Statistical significance for non-inferiority was not reached for a non-composite outcome of mortality alone, according to preliminary results.

If HDx does wind up showing mortality outcomes similar to high-volume hemodiafiltration, one can make some inferences regarding the importance (or lack thereof) of some of the non-middle-molecule features of hemodiafiltration discussed above. HDx does not involve any extracorporeal circuit cooling. Contrary to studies with hemodiafiltration, there is scant evidence that HDx results in a lower incidence of intradialytic hypotension compared with hemodiafiltration [57]. Although ultrapure dialysate is not officially mandated for use with HDx, in the MOTheR trial, ultrapure dialysate was used. HDx vs. hemodiafiltration comparisons in which ultrapure dialysate was used for both groups cannot inform if non-inferiority of HDx vs. hemodiafiltration will be maintained if HDx is used with standard dialysate. Clearance of small molecules such as urea and phosphate is increased with HDx, to a level similar to that observed with high-volume hemodiafiltration [59]. With HDx, bagged saline continues to be used for initial priming, treatment of intradialytic blood volume contraction, and rinse back. Should the results of HDx and high-volume hemodiafiltration be comparable, it would suggest that avoidance of saline with hemodiafiltration is more of an environmental and cost benefit and does not contribute to better outcomes.

## 8. Overall Summary

This narrative review considers potential mechanisms other than enhanced middle-molecule removal whereby survival may be increased in patients treated by hemodiafiltration compared with those treated by hemodialysis. While each of these mechanisms might contribute only partially to observed survival benefits, collectively, they may have an impact.

## Figures and Tables

**Table 1:** Mean predialysis serum beta-2-microglobulin (β2-M) values as reported in four of the five major randomized comparisons of hemodiafiltration (HDF) with hemodialysis (HD).

Study First Author, (Study Nickname), Ref.	N of Cases HDF/HD	Baseline Predialysis Serum β2-M (mg/L) (HDF)	Follow-Up Predialysis Serum β2-M (mg/L) (HDF)	Baseline Predialysis Serum β2-M (mg/L) (HD)	Follow-Up Predialysis Serum β2-M (mg/L) (HD)
Grooteman (CONTRAST) [1]	358/356	30.7	26.4	32.3	35.4
Ok (TURKISH) [3]	391/391	26.5	27.1	26.1	27.2
Maduell (ESHOL) [2]	456/450	23.9	25.8	24.8	24.1
Morena (FRENCHIE) [4]	190/191	26.0	25.8	27.7	27.0
Blankestijn (CONVINCE) [5]	683/677	N/A	N/A	N/A	N/A

Follow-up values are those reported 12 months after randomization. In 4/5 trials, postdilution mode HDF was used exclusively. In Morena (FRENCHIE), mixed mode HDF (predilution + postdilution) was used. Average treatment specifications were: CONTRAST: HD (low-flux): Qb 299 mL/min, Qdnot reported; HDF: Qb 302 mL/min, Qd not reported, Substitution fluid flow rate: 92 postdilution mL/min; Ok: HD (high-flux): Qb 294 mL/min, Qd 500 mL/min; HDF: Qb 294 mL/min, Qd 500 mL/min, Substitution fluid flow rate: 72 postdilution mL/min; ESHOL: HD (high-flux): Qb 380 mL/min, Qd 531 mL/min; HDF: Qb 392 mL/min, Qd 553 mL/min, Substitution fluid flow rate: 102 postdilution mL/min; Morena: HD (high-flux): Qb 335 mL/min, Qd 509 mL/min; HDF: Qb 338 mL/min, Qd 539 mL/min, (Follow-up) substitution fluid flow rate: 172 predilution/88 postdilution mL/min;Blankestijn: HD (high-flux): Qb 367 mL/min, Qd not reported; HDF: Qb 369 mL/min, Qd not reported, Substitution fluid flow rate: 105 postdilution mL/min.

**Table 2:** Kt/V urea values as reported in five major randomized comparisons of hemodiafiltration (HDF) with hemodialysis (HD).

Study First Author, (Study Nickname), Ref.	N of Cases HDF/HD	Baseline Kt/V Urea (HDF)	Follow-Up Kt/V Urea (HDF)	Baseline Kt/V Urea (HD)	Follow-Up Kt/V Urea (HD)
Grooteman (CONTRAST) [1]	358/356	1.41 (sp)	1.63 (sp)	1.38 (sp)	1.45 (sp)
Ok (TURKISH) [3]	391/391	1.44 (e)	1.44 (e)	1.42 (e)	1.33 (e)
Maduell (ESHOL) [2]	456/450	1.67 (sp)	1.83 (sp)	1.66 (sp)	1.73 (sp)
Morena (FRENCHIE) [4]	190/191	1.62 (sp)	1.69 (sp)	1.56 (sp)	1.54 (sp)
Blankestijn (CONVINCE) [5]	683/677	1.61 (sp)	1.74 (sp)	1.61 (sp)	1.65 (sp)

Follow-up values are those reported 12 months after randomization. sp, single-pool; e, equilibrated. For treatment specifications see the legend to Table 1.

## Data Availability

No new data were created or analyzed in this study. Data sharing is not applicable to this article.
